# NAT10 Regulates LPS-Induced Inflammation via Stabilization of N4-Acetylated PTX3 mRNA in Human Dental Pulp Stem Cells

**DOI:** 10.3390/ijms26094325

**Published:** 2025-05-02

**Authors:** Zihan Ni, Luhui Cai, I-Chen Tsai, Wenqian Ding, Cheng Tian, Di Li, Qiong Xu

**Affiliations:** 1 Hospital of Stomatology, Sun Yat-sen University, 56# Lingyuan West Road, Guangzhou 510055, China; nizh3@mail2.sysu.edu.cn (Z.N.); cailh8@mail.sysu.edu.cn (L.C.); tsaiic@mail2.sysu.edu.cn (I.-C.T.); dingwq3@mail2.sysu.edu.cn (W.D.); tianch6@mail.sysu.edu.cn (C.T.); lidi25@mail.sysu.edu.cn (D.L.); 2 Guangdong Provincial Key Laboratory of Stomatology, Guangzhou 510055, China; 3 Guanghua School of Stomatology, Sun Yat-sen University, Guangzhou 510055, China

**Keywords:** N-acetyltransferase 10 (NAT10), lipopolysaccharide (LPS), inflammation, N4-acetylcytidine (ac^4^C), human dental pulp stem cells (hDPSCs), pentraxins 3 (PTX3)

## Abstract

Severe dental pulp inflammation can lead to tissue lysis and destruction, underscoring the necessity for effective treatment of pulpitis. N-acetyltransferase 10 (NAT10)-mediated N4-acetylcytidine (ac^4^C) modification has recently emerged as a key regulator in inflammatory processes. However, whether NAT10 affects the inflammatory response in human dental pulp stem cells (hDPSCs) remains unelucidated. In this study, elevated NAT10 expression was observed in pulpitis tissues and LPS-stimulated hDPSCs. Knockdown of NAT10 led to reduced inflammatory gene expression and lower reactive oxygen species (ROS) production in LPS-stimulated hDPSCs, while the chemotactic migration of macrophages was also suppressed. Similar results were observed when hDPSCs were treated with Remodelin, an inhibitor of NAT10. Differentially expressed genes identified through RNA sequencing were significantly enriched in inflammatory signaling pathways after NAT10 depletion. Among the differential genes, pentraxins 3 (PTX3) was identified as the potential target gene due to the presence of the ac^4^C modification site and its known ability to regulate dental pulp inflammation. The mRNA and protein levels of PTX3 were reduced in NAT10-deficient cells, along with a decrease in its mRNA stability. Exogenous PTX3 supplementation partially reversed the inflammatory inhibition induced by NAT10 knockdown. Further evidence in vivo revealed that Remodelin treatment attenuated the severity of dental pulp inflammation in rats with pulpitis. In summary, these data indicated that NAT10 deficiency inhibited the stability of PTX3 mRNA and further inhibited hDPSC inflammation, while Remodelin might be a potential therapeutic agent for pulp capping.

## 1. Introduction

Pulpitis is a common oral disease, with dental caries and tooth trauma as the main causes [1]. Without medical intervention, dental pulp inflammation may further progress to irreversible pulpitis and necrosis, leading to intolerable pain and potential tooth loss, which will significantly affect the quality of life [2]. The inflammatory response in the dental pulp is typically triggered by the invasion of microorganisms, particularly Gram-negative bacteria, which release lipopolysaccharide (LPS) as a key pathogenic component [3,4]. LPS has been extensively studied for its ability to induce inflammation of various cell types in dental pulp, including human dental pulp stem cells (hDPSCs), making it a valuable tool for simulating the microenvironment of pulpitis in vitro and in vivo [5,6,7]. DPSCs, as progenitor cells within the dental pulp, possess remarkable multidirectional differentiation potential, playing a critical role in pulp tissue homeostasis [8]. In the initial stage of dental pulp inflammation, DPSCs migrate to inflammatory sites and differentiate into odontoblast-like cells, facilitating reparative dentin formation and blocking bacterial invasion [9]. However, further development of inflammation limits the function of DPSCs, suggesting that timely and effective control of inflammation is essential for the repair and regeneration of dental pulp [10,11,12]. Studies have shown that nuclear factor κB (NF-κB) and mitogen-activated protein kinase (MAPK) signaling pathways are critical in the pathogenesis and development of DPSC inflammatory response [13,14]. Inhibition of the signaling activation can limit the synthesis and release of pro-inflammatory molecules, thus avoiding the aggravation of inflammation and protecting dental pulp from damage [15,16,17].

Accumulating studies demonstrate that epigenetic modification participates in multiple physiological processes [18,19,20]. The newly characterized mRNA modification, N4-acetylcytidine (ac^4^C), is crucial for modulating both mRNA stability and the efficiency of translation [21]. N-acetyltransferase 10 (NAT10), the sole identified ac^4^C acetyltransferase, is considered to be vital in various cellular biological processes, including periodontal stem cell osteogenic differentiation, gastric cancer cell metastasis, and multiple myeloma cell apoptosis [22,23,24]. Recently, emerging evidence suggests that NAT10-mediated ac^4^C modification is associated with inflammatory responses. Notably, the effects of NAT10 may vary in different cell types. In neutrophils, NAT10 has been reported as an important negative regulator of pyroptosis; downregulation of NAT10 exacerbates sepsis by increasing pyroptosis in neutrophils via the ULK1-STING-NLRP3 axis [25]. Inconsistent with this, a previous study found that overexpression of NAT10 aggravated LPS-induced inflammation through the NOX2-ROS-NF-κB axis in macrophages, and Remodelin, a specific inhibitor of NAT10, alleviated macrophage infiltration in mice with periodontitis [26]. While these results indicated that the regulatory role of NAT10 in inflammation may be cell-specific, whether and how NAT10 modulates inflammation in hDPSCs has not been elucidated.

In this study, the inflammatory model of hDPSCs in vitro and the pulpitis model of rats in vivo were constructed. By knocking down NAT10 and using its inhibitor Remodelin, this study investigated the regulatory effect and potential mechanism of ac^4^C modification mediated by NAT10 in the regulation of pulpitis.

## 2. Results

### 2.1. Expression of NAT10 Is Upregulated in Inflamed Dental Pulp and LPS-Treated hDPSCs

**Figure 1:**
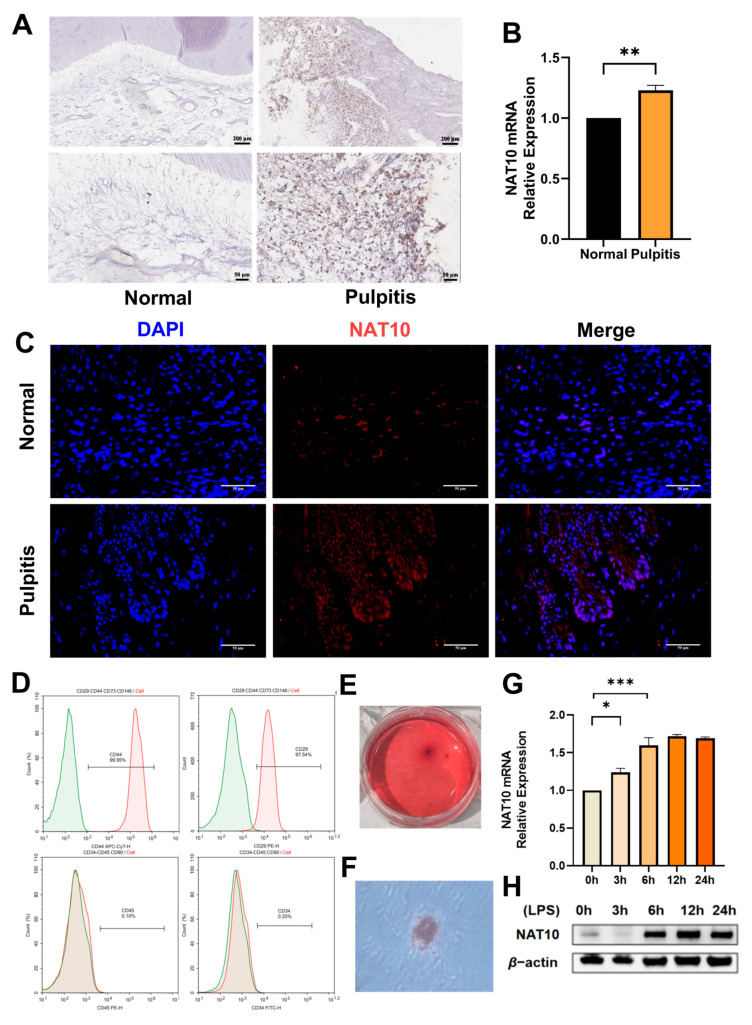
NAT10 expression in inflamed human dental pulp and LPS-stimulated dental pulp stem cells. (**A**,**B**) NAT10 expression in human dental pulp from healthy and inflamed teeth was evaluated using immunohistochemistry (bar = 200 μm for the upper row and bar = 50 μm for the lower row) and qRT-PCR. (**C**) Immunofluorescence was used to assess NAT10 expression in rat dental pulp from control group and pulpitis group (bar = 50 μm). (**D**) The surface markers of hDPSCs (CD44, CD29, CD45, and CD34) were identified by flow cytometry. (**E**,**F**) Multidirectional differentiation potential of hDPSCs was assessed via ARS (**E**) and Oil Red O staining (**F**). (**G**,**H**) hDPSCs were exposed to lipopolysaccharide (LPS, 1 μg/mL) for specific time points. (**G**) NAT10 mRNA levels were quantified via qRT- PCR. (**H**) Western blot was performed to assess NAT10 protein expression. β-actin served as the internal control to normalize qRT-PCR and Western blot data. Results are presented as mean ± SD (n = 3). * *p* < 0.05, ** *p* < 0.01, *** *p* < 0.001.

To determine the involvement of NAT10 in dental pulp inflammation, immunohistochemistry was conducted on healthy and inflamed human dental pulp (Figure 1A). The results showed that NAT10 expression was elevated in pulpitis tissues compared to healthy pulp tissues, which was in line with the expression of NAT10 mRNA (Figure 1B). Similar results were obtained when NAT10 expression was detected in rat dental pulp from the control group and pulpitis group by immunofluorescence (Figure 1C). hDPSCs were cultured from human dental pulp and identified through flow cytometric analysis of surface protein markers and an assessment of their multi-directional differentiation capacity (Figure 1D–F); the expression of NAT10 was then evaluated in LPS-treated hDPSCs in vitro. The data from qRT-PCR (Figure 1G) and Western blot (Figure 1H) revealed that LPS treatment caused a time-dependent increase in NAT10 expression, implying a putative role of NAT10 in modulating inflammation in hDPSCs.

### 2.2. NAT10 Knockdown Attenuates LPS-Induced Inflammation in hDPSCs

**Figure 2:**
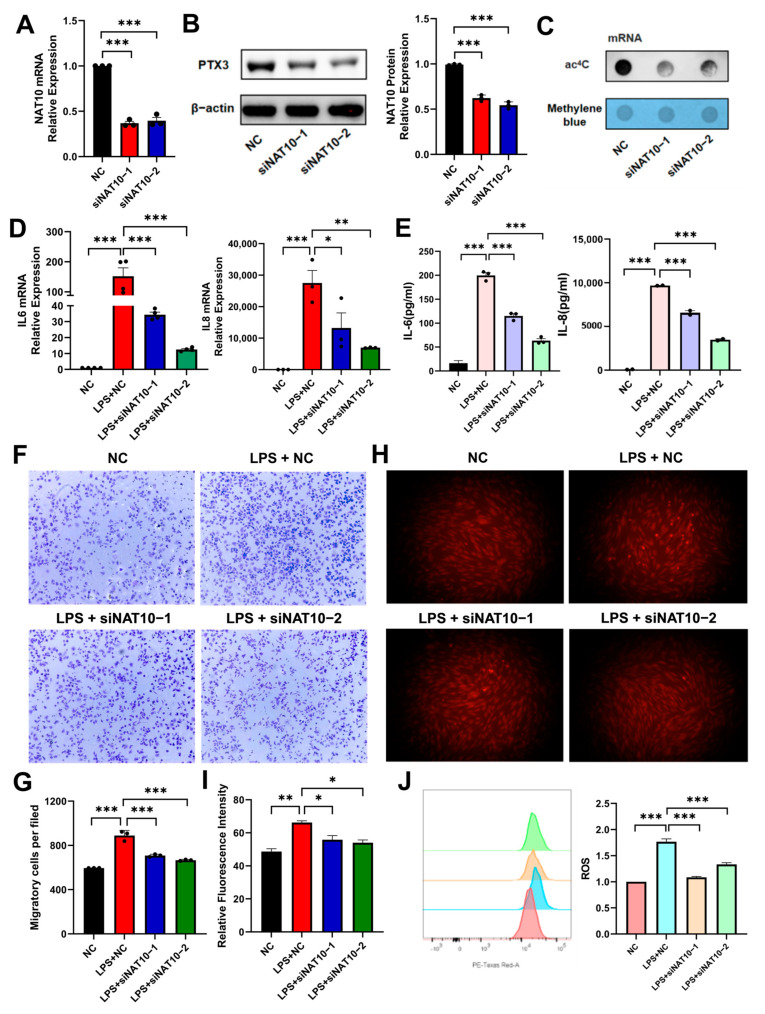
Effect of NAT10 knockdown on LPS-induced inflammation in hDPSCs. (**A**) NAT10 mRNA expression was determined via qRT-PCR in siNAT10 hDPSCs. (**B**) NAT10 protein level was assessed by Western blot. (**C**) The ac^4^C content was determined by dot blot. (**D**) qRT-PCR was used to quantify IL-6 and IL-8 mRNA levels, markers of inflammation. (**E**) The expression of IL-6 and IL-8 proteins was assessed via ELISA. (**F**,**G**) The migration of macrophages was determined by transwell assay. (**H**–**J**) Immunofluorescence and flow cytometry were utilized to quantify ROS levels in NAT10-deficient cells after LPS treatment. Results are presented as mean ± SD (n = 3). * *p* < 0.05, ** *p* < 0.01, *** *p* < 0.001.

To explore the potential role of NAT10 in the inflammatory regulation of hDPSCs, siRNA transfected with plasmid was used to inhibit the expression of NAT10. NAT10 mRNA and protein expression significantly decreased in the siNAT10 groups (Figure 2A,B). Meanwhile, the ac^4^C level of mRNA was also reduced (Figure 2C), validating the effectiveness of NAT10 knockdown. NAT10-silenced hDPSCs were then stimulated with LPS to induce inflammation. The siNAT10 groups exhibited decreased mRNA and protein levels of the inflammatory cytokines, IL-6 and IL-8, compared to the negative control (NC) group (Figure 2D,E). This study then explored whether the chemotactic migration in macrophages can be inhibited by knocking down NAT10 in hDPSCs. As expected, the transwell assay demonstrated that NAT10-deficient hDPSCs in the lower chamber of transwells suppressed the chemotactic migration of macrophages from the upper to the lower chambers (Figure 2F,G). In addition, immunofluorescence and flow cytometry displayed decreased ROS production in the siNAT10 groups (Figure 2H–J), which also reflected suppressed inflammation.

### 2.3. Inhibition of NAT10 Represses LPS-Activated NF-Kappa B and MAPK Signaling Pathways in hDPSCs

**Figure 3:**
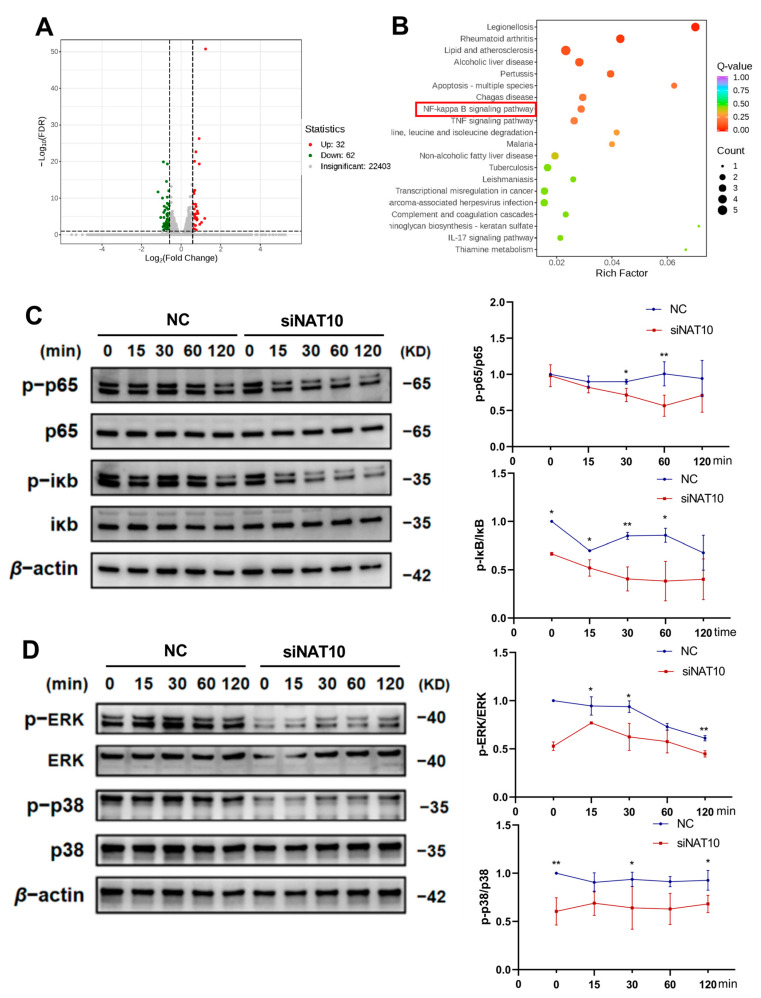
Effect of NAT10 deficiency on activation of NF-kappa B and MAPK signaling pathways in LPS-stimulated hDPSCs. (**A**) Differentially expressed genes (DEGs) between the siNAT10 and NC groups were exhibited using volcano plots, with Q value < 0.05 indicating significance. (**B**) Enriched KEGG pathways of DEGs. (**C**,**D**) Western blot was performed to quantify the phosphorylation levels of IκB, p65, ERK, and p38. Results are presented as mean ± SD (n = 3). Results are presented as mean ± SD (n = 3). * *p* < 0.05, ** *p* < 0.01.

To elucidate the underlying molecular mechanisms of NAT10 in regulating hDPSC inflammation, RNA sequencing (RNA-seq) analysis was conducted to investigate the effects of NAT10 depletion (Figure 3A). KEGG pathway analysis indicated significant enrichment of differentially expressed genes in inflammatory response pathways, notably the NF-κB signaling pathway (Figure 3B). Western blot analysis confirmed that siNAT10 cells exhibited reduced NF-κB pathway activity, as evidenced by decreased Iκ B and p65 phosphorylation (Figure 3C). The MAPK signaling pathway was also evaluated, and the data showed that NAT10 depletion resulted in decreased phosphorylation of ERK and p38 (Figure 3D). These results indicated that LPS-induced activation of NF-κB and MAPK signaling in hDPSCs was suppressed by NAT10 knockdown.

### 2.4. PTX3 May Be an ac^4^C -Modified Target of NAT10 in hDPSC Inflammation

**Figure 4:**
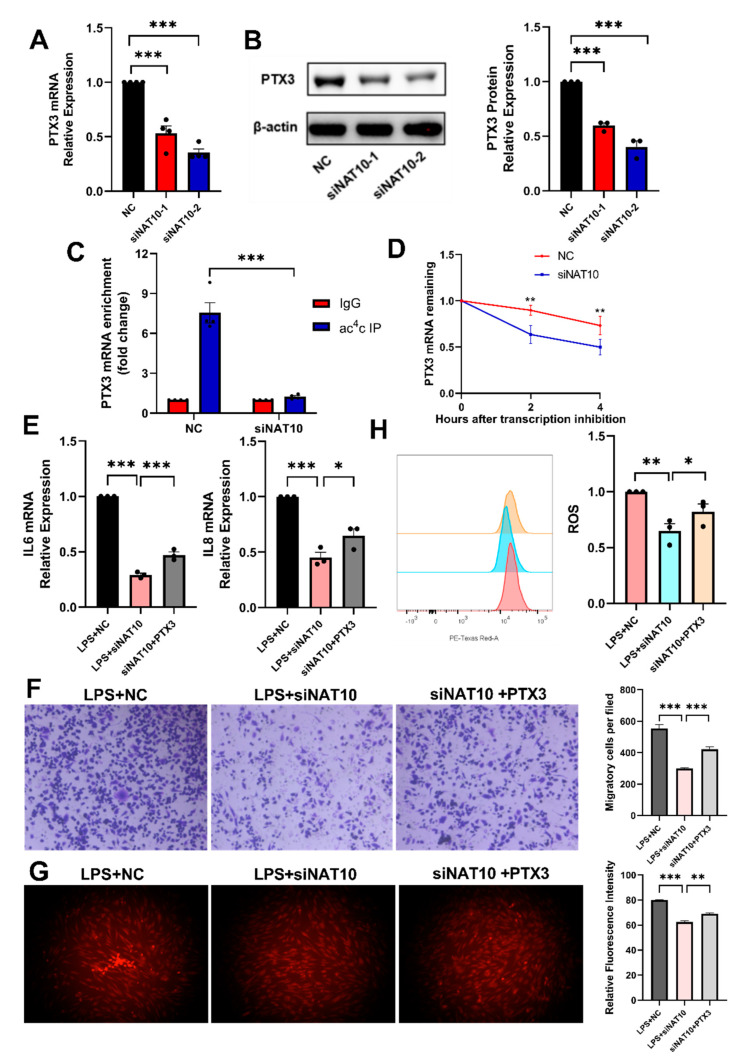
Identification of PTX3 as a target gene for the inflammatory response in NAT10-deficient cells. (**A**) The mRNA expression of PTX3 was analyzed by qRT-PCR after NAT10 knockdown. (**B**) PTX3 protein level was assessed using Western blot. (**C**) RIP-PCR analysis was performed with IgG antibody and anti-ac^4^C antibody to detect the effect of knocking down NAT10 on PTX3 expression level. (**D**) Actinomycin D treatment of hDPSCs was followed by qRT-PCR analysis to quantify PTX3 mRNA expression. (**E**) The mRNA levels of IL-6 and IL-8 were quantified by qRT-PCR. (**F**) Macrophage migration was evaluated via transwell assay. (**G**,**H**) The ROS level was measured using immunofluorescence and flow cytometry. Results are presented as mean ± SD (n = 3). * *p* < 0.05, ** *p* < 0.01, *** *p* < 0.001.

Sequencing analysis revealed reduced PTX3 expression, which was further confirmed by lower PTX3 mRNA and protein expression in the siNAT10 groups (Figure 4A,B). The ac^4^C enrichment in PTX3 was also decreased after NAT10 knockdown, as shown in acRIP-qRCR (Figure 4C). Following Actinomycin D treatment, PTX3 mRNA stability decreased upon NAT10 depletion, implying the role of acetylation in maintaining PTX3 mRNA stability (Figure 4D). To validate whether the inflammatory suppression induced by NAT10 knockdown was achieved by inhibiting PTX3, NAT10-knockdown cells were supplied with human recombinant PTX3 (rmPTX3) to promote its expression. As the results showed, the decreased expression levels of inflammatory cytokine were partially reversed by rmPTX3 (Figure 4E). Furthermore, rmPTX3 also partially restored the inhibition of macrophage chemotactic migration and ROS production induced by NAT10 knockdown (Figure 4F–H). In summary, these data indicated that NAT10 might regulate hDPSC inflammation by catalyzing ac^4^C modification of PTX3 mRNA.

### 2.5. Remodelin Alleviates hDPSC Inflammation Induced by LPS and Dental Pulp Inflammation of Rats with Pulpitis

**Figure 5:**
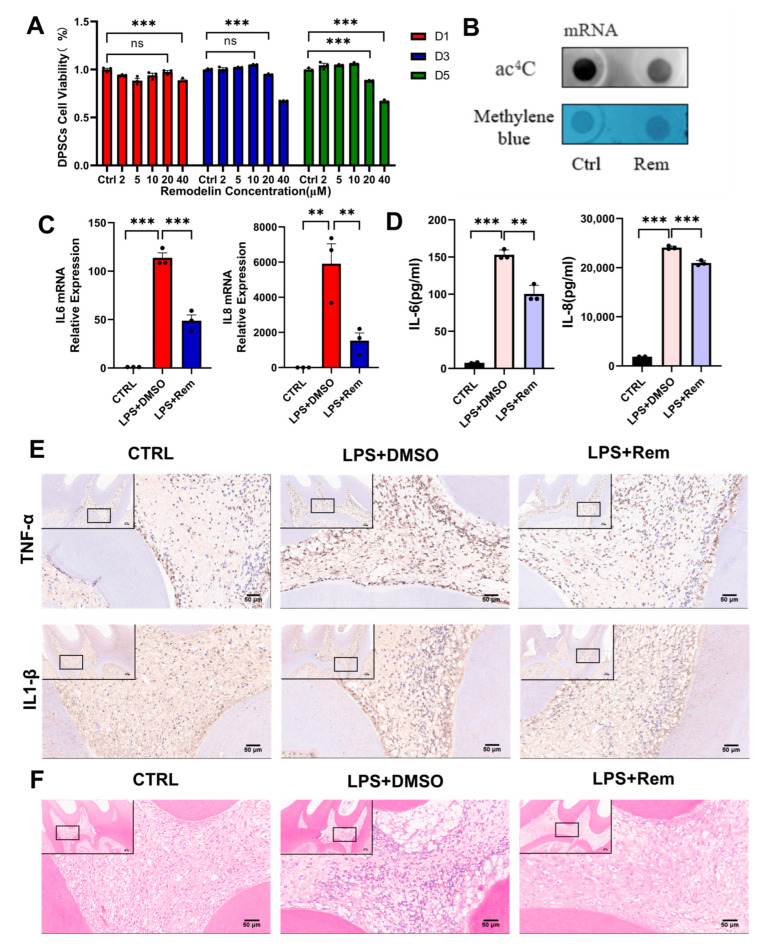
The anti-inflammatory effect of Remodelin in vitro and in vivo. (**A**) hDPSC viability was determined via CCK8 assay. (**B**) Dot blot was used to measure the ac^4^C content. (**C**) qRT-PCR was used to quantify IL-6 and IL-8 mRNA levels. (**D**) The expression of IL-6 and IL-8 proteins was assessed via ELISA. (**E**) Representative images of IHC staining of TNF-α and IL-1β in rat dental pulp tissue sections. (**F**) Representative images of H&E-stained paraffin slices. Results are presented as mean ± SD (n = 3). ** *p* < 0.01, *** *p* < 0.001, ns, not significant.

Remodelin, a specific inhibitor of NAT10, was evaluated for its therapeutic potential in the inflammatory model of hDPSCs in vitro and the pulpitis model of rats in vivo. To maintain the viability of hDPSCs, Remodelin with a concentration of 10μM was chosen, as suggested by a CCK8 assay (Figure 5A). After treatment with Remodelin, the expression of ac^4^ C modification was downregulated, indicating the inhibition of acetylation (Figure 5B). qRT-PCR and ELISA analyses revealed a significant decrease in IL-6 and IL-8 expression in the Remodelin-treated group compared to the control group, suggesting that LPS-induced hDPSC inflammation was attenuated by Remodelin (Figure 5C,D). The effect of Remodelin on rats with pulpitis in vivo was further investigated. Immunohistochemistry demonstrated that Remodelin treatment significantly suppressed the expression of pro-inflammatory cytokines TNF-α and IL-1β in the inflamed dental pulp tissue, relative to the LPS + DMSO group (Figure 5E). Meanwhile, H&E staining exhibited a marked reduction in inflammatory cell infiltration within the dental pulp of rats treated with Remodelin (Figure 5F). These results suggested a potential anti-inflammatory effect of Remodelin in the treatment of pulpitis.

## 3. Discussion

Although inflammatory response is a prerequisite for pulp healing and regeneration, severe or persistent inflammation can be detrimental [27,28]. Lipopolysaccharide (LPS), a well-established inflammatory stimulus, was chosen for this study due to its ability to mimic the pathological conditions of pulpitis by activating immune cells and dental pulp cells to produce a spectrum of inflammatory cytokines [29]. This makes LPS an ideal agent for investigating the molecular mechanisms underlying dental pulp inflammation. In this study, an inflammatory environment in vitro was induced by stimulating hDPSCs with LPS, in which increased NAT10 expression was observed.

Studies of ac^4^C modification initially focused on tRNA and 18S rRNA; however, it is now considered a widespread modification in the human transcriptome [30]. NAT10, the sole identified ac^4^C “writer” protein, plays a critical role in mRNA stabilization and translation by ensuring accurate decoding of specific nucleotide sequences [31]. Emerging evidence links NAT10-mediated ac^4^C modification to the pathogenesis of numerous disorders like gastric cancer, osteoporosis, and cardiac fibrosis [32,33]. In particular, the impact of NAT10 on inflammation, as revealed by recent studies, has broadened the understanding of its role in pathogenicity and underlying mechanisms of disease [34,35,36]. Nevertheless, the regulation of NAT10 on inflammation of different cell types is not yet conclusive, and its potential involvement in modulating hDPSC inflammation has not been reported.

In the present study, increased NAT10 expression was observed in pulp tissues from patients with pulpitis, which is consistent with the expression of NAT10 in LPS-stimulated hDPSCs. NAT10′s regulatory effect on hDPSC biological behavior was then investigated through NAT10 knockdown experiments, which revealed a significant downregulation of key inflammatory mediators, IL-6 and IL-8. Previous studies have demonstrated that dental pulp fibroblasts may influence the progression of pulpitis by regulating the polarization and localization of macrophages [37]. Specifically, it has been shown that the supernatant of LPS-stimulated dental pulp cells increases the number of recruited macrophages, suggesting that macrophages can migrate to the site of dental pulp inflammation [1,38]. The present data showed that knocking down NAT10 remarkably inhibited macrophage migration, which reflected the inhibitory effect of NAT10 knockdown on hDPSC inflammation. The observed reduction in ROS levels following NAT10 knockdown provides additional mechanistic insights, as ROS generation is not only a byproduct of mitochondrial oxidative metabolism but also a critical component of immune responses in inflammatory diseases [39,40]. The decreased ROS expression in our study suggests that NAT10 knockdown suppresses inflammation in LPS-stimulated hDPSCs through multiple pathways, including modulation of cytokine expression, macrophage migration, and oxidative stress responses.

To explore the potential mechanisms by which NAT10 regulates inflammation in hDPSCs, RNA sequencing was performed with and without NAT10 knockdown. KEGG analysis identified significant enrichment of differentially expressed genes in the NF-κB pathway, a classical inflammatory reaction pathway; the MAPK signaling pathway was also tested for its role in hDPSC inflammation modulation [41,42], despite no enrichment found in the sequencing. As verified by Western blot, knocking down NAT10 inhibited the phosphorylation of key components in NF-κB and MAPK signaling pathways.

Pentraxin 3 (PTX3), a soluble pattern recognition molecule, belongs to an evolutionarily conserved superfamily of soluble pattern recognition molecules in the innate immune system [43]. As a multifunctional protein, PTX3 has been shown to be actively involved in cardiovascular diseases, tumorigenesis, and inflammatory disorders [44,45,46]. Notably, according to Kim et al., the expression of PTX3 was elevated in TNF-α-treated dental pulp cells, and PTX3 knockdown led to a substantial downregulation of IL-6 and IL-8, indicating the regulatory role of PTX3 in pulpitis [47]. Other than that, Liu et al. characterized PTX3 as the direct target of NAT10 through ac^4^C-seq and RNA-seq in rheumatoid arthritis (RA) fibroblast-like synoviocytes (FLSs); the mutant of PTX3 engineered to replace the ac^4^C consensus sequence CXX with GXX proved that the modification of ac^4^C mediated by NAT10 directly regulated PTX3 expression [48]. Together with the role of PTX3 in regulating the inflammation of dental pulp cells, it can be reasonably inferred that PTX3 may be a target gene for NAT10 to regulate inflammation in hDPSCs. In this study, the inhibition of inflammation caused by NAT10 knockdown was accompanied by a decreased PTX3 expression level, and the provision of exogenous PTX3 partially reversed the effect of NAT10 knockdown on inflammation. It was further demonstrated that ac^4^C acetylation mediated by NAT10 regulated the expression of PTX3 by stabilizing its mRNA. While this study provides evidence for PTX3 as the target of NAT10 in hDPSC inflammation regulation, the involvement of other unidentified proteins acting as potential NAT10 targets in this process cannot be ruled out. Further studies aimed at identifying and characterizing these additional targets will be essential to fully elucidate the mechanistic complexities of NAT10-mediated inflammation and to refine therapeutic approaches for inflammatory conditions in dental pulp and beyond.

To further investigate the therapeutic potential of Remodelin, a specific NAT10 inhibitor, we applied it to a LPS-stimulated DPSC and pulpitis model of rats. Results suggested that Remodelin treatment downregulated inflammatory factor expression in LPS-treated hDPSCs. Furthermore, Remodelin significantly lowered TNF-α and IL-1β protein levels in the dental pulp of rats with pulpitis, concurrently reducing inflammatory cell infiltration within the dental pulp tissue. These results indicated that NAT10-mediated ac^4^C modification may represent a viable therapeutic target for pulpitis, and Remodelin exhibited the potential as a pulp capping agent. For in vivo experiments, rats were selected due to the similarity of their tooth anatomy and cellular processes to those of humans [49]. However, it is important to note that there are physiological and metabolic distinctions between rats and humans, which is a potential limitation of this study. These interspecies differences may influence the interpretation and translational relevance of the results.

In conclusion, this study showed that NAT10 expression was elevated in inflamed human dental pulp and LPS-stimulated hDPSCs. Both NAT10 knockdown and pharmacological inhibition with Remodelin effectively attenuated hDPSC inflammation. Mechanistically, NAT10 regulates cell inflammation by promoting the mRNA stability of N4-acetylated PTX3. In vivo experiments further validated these findings, as Remodelin treatment markedly alleviated the severity of dental pulp inflammation in rats with pulpitis. Collectively, these findings revealed the potential role of RNA epigenetic modification in regulating hDPSC inflammatory response, and Remodelin demonstrated promising characteristics as a pulp capping agent, warranting further investigation into its clinical application.

## 4. Materials and Methods

### 4.1. Collection of Clinical Samples

This research was conducted with ethical approval from the Ethics Committee of the Affiliate Stomatology Hospital of Sun Yat-sen University (KQEC-2025-035-01). All participants gave written informed consent. The dental pulp tissues in this study were extracted from premolars and third molars extracted for orthodontic or surgical purposes. Samples were categorized into healthy and pulpitis groups based on clinical presentation.

### 4.2. Culture of Primary Human Dental Pulp Stem Cells (hDPSCs)

Healthy human dental pulp tissues were obtained from people aged 18–25 years old. Cell suspensions were generated by digesting washed and minced tissues with collagenase I (Sigma-Aldrich, St. Louis, MO, USA). The suspension was subsequently filtered and centrifuged before being cultured in α-MEM (Gibco, New York, NY, USA) with 20% FBS (Cellmax, Lanzhou, China) and 1% penicillin-streptomycin (NCM, Suzhou, China) supplementation. Cells at passages 3–5 were used throughout the study.

### 4.3. Induction of Osteogenic and Adipogenic Differentiation of hDPSCs

The culture medium, comprising α-MEM with the addition of 50 μg/mL ascorbic acid, 10 mM β-glycerophosphate, and 100 nM dexamethasone (Sigma-Aldrich, St. Louis, MO, USA), was used to induce osteogenic differentiation of hDPSCs. hDPSCs were cultured for 7 or 21 days. Adipogenic differentiation of hDPSCs was induced by α-MEM supplemented with 500 μM IBMX, 10 μg/mL insulin, 100 μM indomethacin, and 1 μM dexamethasone. The culture of hDPSCs was sustained for 21 days.

### 4.4. Alizarin Red (ARS) and Oil Red O Staining

Following fixation in 4% paraformaldehyde, cells were subjected to specific staining protocols. Calcium deposits were visualized using 1% Alizarin Red staining (GL Biochem, Shanghai, China). Lipid droplet formation was tested through Oil Red O staining. All stained cells were subsequently photographed using an inverted phase-contrast microscope (IX83 + DP74, Olympus, Tokyo, Japan).

### 4.5. Flow Cytometric Analysis

For cell surface marker analysis, 5 × 10^5^ hDPSCs were collected, suspended in 75 μL PBS, and then incubated in darkness with fluorochrome-conjugated antibodies against human CD34, CD45, CD29, CD44, and IgG. After incubation, hDPSCs were prepared for flow cytometric analysis by washing with 800 μL PBS via centrifugation (300× *g*, 5 min), followed by resuspension in 500 μL PBS. Cellular analysis was performed using a flow cytometer (BD Biosciences, Franklin Lakes, NJ, USA).

### 4.6. Immunohistochemistry

The healthy and inflamed human dental pulp was processed for immunohistochemical analysis. Following dehydration and paraffin embedding, 5 μm sections were prepared. These sections underwent deparaffinization, rehydration, and antigen retrieval in a citric acid buffer. The following steps were conducted using the Streptavidin-Peroxidase kit (ZSGB-Bio). Sections were incubated overnight in primary antibody solution against NAT10 (1:250; ABclonal, Wuhan, China) at 4 °C. DAB was used for staining, with an approximate incubation time of 20 s. Hematoxylin was applied as a counterstain for 10 s. Nuclear staining ranging from light yellow to brown was considered indicative of NAT10 positivity. The sections were scanned with a slice scanner (Leica Biosystems, Buffalo Grove, IL, USA).

### 4.7. Cell Proliferation Assay

hDPSCs cultured in 96-well plates were exposed to stimuli for designated periods. The viability was detected via a CCK-8 assay (GLPBIO, Montclair, CA, USA) by measuring absorbance at 450 nm.

### 4.8. Cell Transfection

A total of 50 nM NAT10 specific siRNAs or a nonspecific (Hippobio, Huzhou, China) and Lipofectamine™ 3000 (Invitrogen, Carlsbad, CA, USA) were transfected into hDPSCs following the protocol to establish knockdown cell lines. The knockdown efficiency was measured via qRT-PCR and Western blot. The siRNA sequences are detailed in Table 1.

### 4.9. Real-Time Quantitative Polymerase Chain Reaction (qRT-PCR)

RNA isolation was performed using RNAzol (MRC, Cincinnati, OH, USA). Subsequently, ABScript III RT Master Mix was employed to generate cDNA (ABclonal, Wuhan, China). A LightCycler 480 (Roche, Basel, Switzerland) was used to conduct qPCR. The primers used in this study are listed in Table 2.

### 4.10. Western Blot

Cells were lysed for 30 min on ice using RIPA buffer (Beyotime, Haimen, China) containing protease and phosphatase inhibitors (Cwbiotech, Beijing, China). Protein concentrations were evaluated by a BCA kit (Cwbiotech, Beijing, China); 25 μg of protein per lane were separated by 10% SDS-PAGE. Polyvinylidene fluoride membranes (Millipore, Billerica, MA, USA), onto which proteins had been transferred, were blocked with 5% bovine serum albumin for 1 h. Next, primary antibodies against NAT10, PTX3 (1:1000; ABclonal, Wuhan, China), ERK, p-ERK, p38, p-p38, p65, p-p65, IκB, p-IκB, (1:800; Cell Signalling Technology, Boston, MA, USA), and β-actin (1: 50000; ABclonal, Wuhan, China), together with the membranes, were held at 4 °C for an overnight period. Proteins were observed by an enhanced chemiluminescence kit (Millipore, Billerica, MA, USA) following secondary antibody incubation.

### 4.11. Enzyme-Linked Immunosorbent Assay (ELISA)

The concentration of IL-6 and IL-8 in hDPSC culture supernatant was analyzed via ELISA kits (ABclonal, Wuhan, China) as the recommended procedure.

### 4.12. Transwell Chemotactic Migration Assay

NAT10 siRNA or a negative control siRNA was used to transfect hDPSCs. Following a 12 h stimulation with LPS, the supernatant was collected. Then, seeded RAW 264.7 cells were placed into the upper compartment of transwell inserts (Corning, NY, USA), with the collected supernatant placed in the lower compartment. Migrated cells, collected from the membrane surface after a 24 h incubation, were fixed using 4% paraformaldehyde and then stained by crystal violet. The cells were subjected to quantification using phase-contrast microscopy (Axiovert 40; Zeiss, Jena, Germany).

### 4.13. Measurement of Reactive Oxygen Species (ROS) Expression

Following LPS stimulation, hDPSCs were incubated with 20 μM DHE (Vigorous, Beijing, China) for 1 h. Flow cytometry (LSRFortessa, Becton Dickinson, Franklin Lakes, NJ, USA) was used to identify labeled cells, and fluorescence emission was analyzed by an inverted fluorescence microscope (Olympus IX83 + DP74, Tokyo, Japan) equipped with a TRITC filter set.

### 4.14. RNA Sequencing

Following total RNA extraction, mRNA was isolated and purified. The resulting RNA samples underwent fragmentation and were subsequently converted into double-stranded cDNA. Illumina sequencing was performed on the cDNA libraries (Metware Biotechnology Co., Ltd., Wuhan, China). Statistically significant differential expression was defined as a *p*-value < 0.05 and |FC| > 2. KEGG pathway enrichment analysis, performed with the BGI platform, was used to explore the functional implications of DEGs.

### 4.15. RNA ac^4^C Dot Blot Assay

Hybond-N+ membranes (Solarbio, Beijing, China) were used to capture mRNA, which had been heat-denatured at 95 °C for 3 min. Crosslinking was performed for 45 min using UV light. The membranes were blocked with 5% BSA for 1 h before overnight incubation with anti-ac^4^C antibody (1:1000; ABclonal, Wuhan, China) at 4 °C. Subsequently, membranes were incubated for 1 h with a 1:2000 dilution of secondary antibody (Cell Signaling Technology, Boston, MA, USA); then, the membranes were observed via the enhanced chemiluminescence kit (Millipore, Billerica, MA, USA). Total input RNA was quantified by staining membranes with 0.2% methylene blue.

### 4.16. RNA-Binding Protein Immunoprecipitation (RIP)

This experiment was conducted using the RIP Kit (Merck Millipore, MA, USA). Cell lysates, prepared from washed cells using RIP lysis buffer, were incubated overnight at 4 °C to bind either an anti-ac^4^C antibody (1:1000, ABclonal, Wuhan, China) or IgG antibody to magnetic beads. Following incubation, beads were washed extensively with PBS to remove non-specifically bound material. Following proteinase K digestion and RNAzol extraction, bound RNA was quantified by qRT-PCR.

### 4.17. Assessment of the mRNA Stability

To assess mRNA stability, global mRNA transcription was inhibited by treating stimulated hDPSCs with actinomycin D (Sigma, St. Louis, MO, USA). RNA was extracted at defined time intervals, and target gene mRNA stability was then determined by measuring relative mRNA levels.

### 4.18. Pulpitis Model in Vivo

The in vivo study was approved by the Animal Care and Use Committee of Sun Yat-sen University (SYSU-IACUC-2024-002551). Eight-week-old male SD rats, weighing 180–250 g, were purchased from the Laboratory Animal Center of Sun Yat-sen University. The rats were randomly divided into three groups (n = 6): the control group, the LPS + DMSO group, and the LPS + Remodelin group. An occlusal cavity was created on the maxillary first molar with a small dental circular drill and an endodontic hand file. The exposed cavity was stimulated with LPS for 15 min to simulate the inflammatory environment, as described in a previous study [50]. The untreated first molar was used as a healthy control. Rats were euthanized after 1 w, and their maxillaries were extracted for subsequent analysis.

### 4.19. H&E, Immunohistochemistry and Immunofluorescence Staining

The maxillaries were fixed in 4% paraformaldehyde at 4 °C for 24 h. Following decalcification in 10% EDTA for two months, samples were paraffin-embedded and sectioned at 4 μm. Hematoxylin and eosin (H&E) and immunohistochemistry staining were performed on deparaffinized and rehydrated sections, adhering to the manufacturer’s recommended procedure. The protocol of immunohistochemistry staining was the same as described above. For immunofluorescence, sections underwent antigen retrieval using a pepsin solution (Servicebio, Wuhan, China). Then, sections were washed with PBS containing 0.1% Triton X-100 (PBST) and blocked with QuickBlock™ (Beyotime, Haimen, China) for 15 min at room temperature. Subsequently, sections were incubated with primary antibodies overnight at 4 °C. After three washes with PBST, secondary antibodies were applied. Finally, cell nuclei were counterstained with DAPI (ABclonal, Wuhan, China). The samples were then analyzed using a fluorescence microscope (FV3000, Olympus, Tokyo, Japan).

### 4.20. Statistical Analysis

Statistical analyses were conducted using GraphPad Prism 9 (GraphPad, San Diego, CA, USA). Data are presented as the mean ± SD. Student’s t-tests or one-way ANOVA were utilized for comparisons. The criterion for statistical significance was set at *p* < 0.05.

## Figures and Tables

**Table 1:** siRNA sequences for NAT10 knockdown.

siRNA	Sequences (5′–3′)
#1 siRNA	GCACCACUGCUGAGAAUAATT
	UUAUUCUCAGCAGUGGUGCTG
#2 siRNA	GCUGCUGCAGAUGUACUAUTT
	AUAGUACAUCUGCAGCAGCTG

**Table 2:** Primer sequences for qRT-PCR.

Gene	Forward Primer (5′–3′)	Reverse Primer (3′–5′)
*IL-6*	CCGGGAACGAAAGAGAAGCTC	ACCGAAGGCGCTTGTGGAG
*IL-8*	TTTCTGATGGAGAGAGCTCTGTCTGG	AGTGGAACAAGACTTGTGGATCCTGG
*PTX3*	CGAAATAGACAATGGACTCCATCC	CTCATCTGCGAGTTCTCCAGCA
*NAT10*	ATAGCAGCCACAAACATTCGC	ACACACATGCCGAAGGTATTG
*β-actin*	CCTGGCACCCAGCACAAT	GGGCCGGACTCGTCATACT

## Data Availability

Data supporting the reported results are available from the corresponding authors to all interested researchers.
